# Developmental defects of enamel and dentin in individuals born preterm: a scoping review of evidence and mechanisms

**DOI:** 10.3389/fdmed.2026.1918126

**Published:** 2026-09-02

**Authors:** Sadhvi B, Manju R, Samiksha Uchil, Rupali D, Swagata Saha

**Affiliations:** 1 Department of Pediatric and Preventive Dentistry, A.B. Shetty Memorial Institute of Dental Sciences, NITTE (Deemed to be University), Karnataka, Mangaluru, India; 2 Dr DY Patil Dental College and Hospital, Dr DY Patil Vidyapeeth University, Pune, India

**Keywords:** dentin defects, developmental defects of enamel, enamel hypomineralisation, enamel hypoplasia, low birth weight, neonatal intensive care unit, odontogenesis, prematurity

## Abstract

**Background:**

Preterm birth (<37 weeks of gestation) occurs during critical stages of odontogenesis and is associated with developmental defects in dental hard tissues. Enamel hypoplasia, enamel hypomineralization, and structural dentin alterations may compromise esthetics, function, and long-term oral health outcomes. However, the nature and extent of these defects and their underlying mechanisms have not been comprehensively investigated.

**Objective:**

To map and evaluate the available evidence regarding developmental defects of enamel and dentin in individuals born preterm, identify proposed pathophysiological mechanisms, and highlight the gaps in the current literature.

**Methods:**

This scoping review was conducted in accordance with the Arksey and O'Malley framework, incorporating methodological refinements proposed by Levac et al., and reported according to the Preferred Reporting Items for Systematic Reviews and Meta-Analyses Extension for Scoping Reviews (PRISMA-ScR). A structured search of PubMed, Scopus, and Web of Science was performed for studies published until July 2026. Studies evaluating enamel and/or dentin outcomes in individuals born preterm (<37 weeks of gestation) were eligible for inclusion.

**Results:**

Fourteen studies, including cross-sectional, cohort, case-control, longitudinal, and histological investigations, met the eligibility criteria. Enamel hypoplasia and hypomineralization were the most frequently reported defects, with greater prevalence and severity observed among individuals with lower gestational age and birth weight. Dentin alterations, including reduced mineralisation, altered hardness, and structural irregularities, were reported in only three of the included studies. Although these findings suggest an association with prematurity, the limited evidence base means that they should be regarded as preliminary and hypothesis-generating rather than conclusive. Proposed mechanisms include perinatal hypoxia, nutritional deficiencies, systemic inflammation, and neonatal intensive care–related interventions, such as endotracheal intubation and prolonged assisted ventilation. The available evidence is characterised by methodological heterogeneity, variations in diagnostic criteria, and a paucity of longitudinal studies.

**Conclusion:**

Current evidence suggests that preterm birth is associated with developmental defects in both enamel and dentin. Nevertheless, significant knowledge gaps remain, particularly regarding dentin-specific pathologies, long-term outcomes, and underlying biological mechanisms. Future research should prioritise standardised diagnostic approaches and well-designed longitudinal studies to strengthen the evidence base and inform preventive and therapeutic strategies for this condition.

## Introduction

Preterm birth, defined as delivery before 37 completed weeks of gestation, remains a major global public health challenge, accounting for approximately 10% of live births worldwide and representing the leading cause of neonatal morbidity and mortality. Advances in neonatal intensive care have substantially improved the survival of preterm and very low birth weight (VLBW) infants; however, increasing survival has also led to greater recognition of the long-term health consequences associated with prematurity (1, 2).

**Figure 1:**
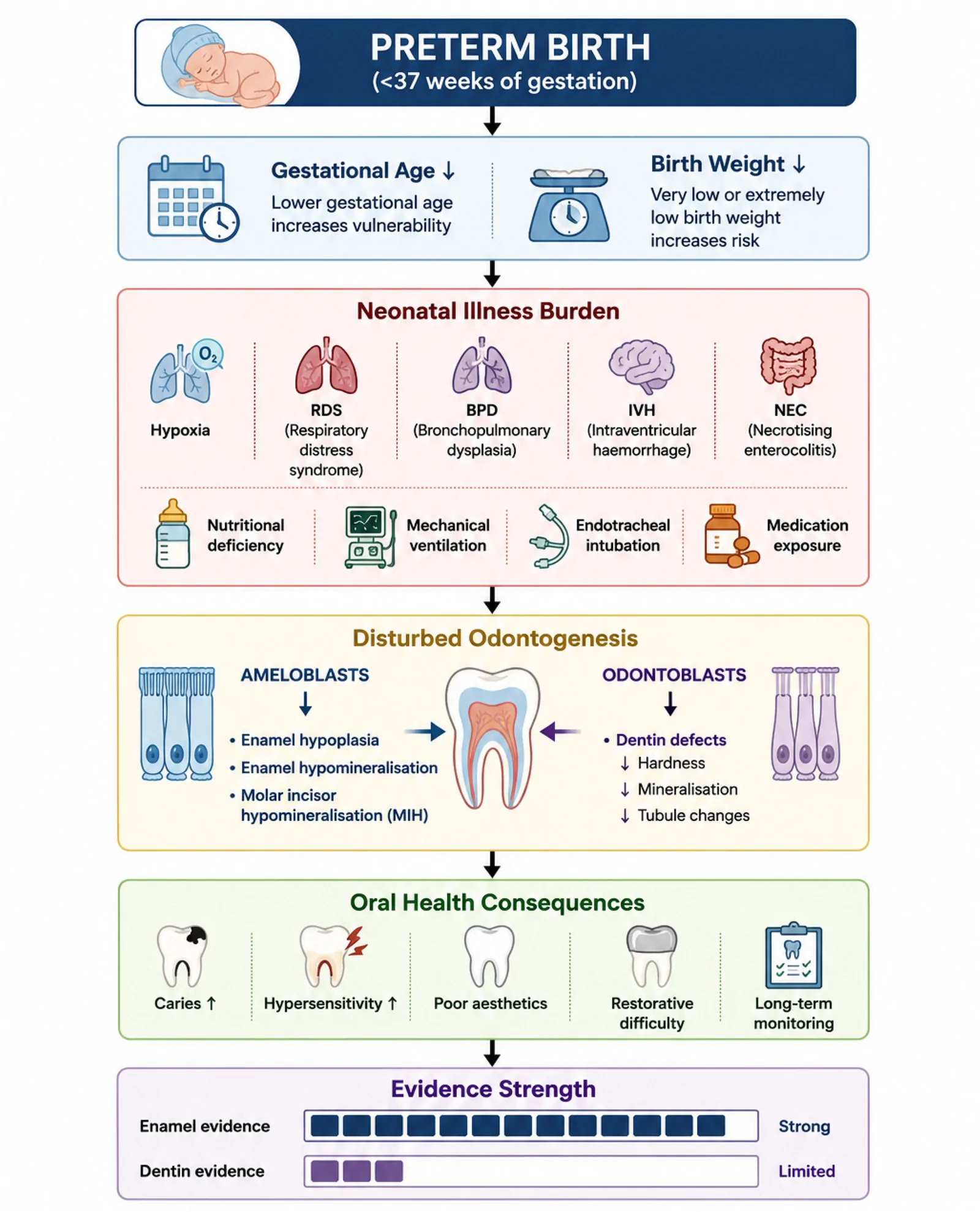
Proposed biological pathways linking preterm birth to developmental defects of enamel and dentin.

Among the less frequently recognised sequelae of preterm birth are developmental defects affecting the dental hard tissues, particularly the enamel and dentin. Odontogenesis is a highly regulated process that begins during intrauterine life and continues into early childhood. The formation and mineralisation of enamel and dentin depend on the coordinated activity of ameloblasts and odontoblasts, which are particularly vulnerable to systemic disturbances during critical developmental periods (3). Preterm infants are commonly exposed to a range of biological and environmental stressors, including perinatal hypoxia, metabolic instability, nutritional deficiencies, systemic infections, and neonatal intensive care interventions, such as endotracheal intubation and prolonged medication exposure (4, 5). These factors may disrupt normal tooth development, resulting in developmental defects in the enamel and dentin (Figure 1).

Developmental defects of the enamel, including enamel hypoplasia and hypomineralisation, have been reported more frequently in preterm and VLBW children than in their full-term counterparts (6). These defects are clinically relevant because they may increase susceptibility to dental caries, dentinal hypersensitivity, structural fragility, aesthetic concerns, and restorative challenges (7, 8). Emerging evidence also suggests that prematurity may affect dentin development, although dentin-related outcomes have received considerably less attention than have enamel defects.

Despite the growing interest in the oral health consequences of prematurity, the existing literature remains fragmented and methodologically heterogeneous. Studies vary substantially with respect to population characteristics, definitions of prematurity, diagnostic criteria, dentition assessed, and outcome measures. Furthermore, previous reviews have primarily focused on enamel defects or specific clinical outcomes, providing limited insights into dentin involvement, underlying biological mechanisms, and broader clinical implications.

Given the diversity of available evidence and the evolving nature of this research field, a scoping review is an appropriate methodology for systematically mapping the extent, range, and characteristics of the literature, identifying key concepts, and highlighting gaps requiring further investigation (9, 10). This review was conducted in accordance with the methodological framework proposed by Arksey and O'Malley, incorporating refinements by Levac et al., and is reported following the Preferred Reporting Items for Systematic Reviews and Meta-Analyses Extension for Scoping Reviews (PRISMA-ScR) guidelines (11, 12).

This scoping review aimed to map and synthesise the available evidence regarding developmental defects of enamel and dentin in individuals born preterm, examine the proposed biological mechanisms underlying these defects, and identify knowledge gaps to inform future research and clinical practice.

## Materials and methods

### Study design and reporting

A scoping review methodology was selected because the available evidence regarding developmental defects of enamel and dentin in individuals born preterm is heterogeneous with respect to study design, populations, diagnostic criteria, and outcome measures. Scoping reviews are particularly suited for mapping the extent, range, and characteristics of evidence, identifying key concepts, and highlighting knowledge gaps within emerging or complex research areas.

This review was conducted in accordance with the methodological framework proposed by Arksey and O'Malley and further refined by Levac et al., which comprises five stages: (1) identification of the research question, (2) identification of relevant studies, (3) study selection, (4) data charting, and (5) collation, summarisation, and reporting of results (9, 10). This review was reported according to the Preferred Reporting Items for Systematic Reviews and Meta-Analyses Extension for Scoping Reviews (PRISMA-ScR) guidelines (11).

### Protocol and registration

A review protocol was developed *a priori* to guide the search strategy, study selection, data charting, and evidence synthesis. Although the protocol was not formally registered, all methodological decisions were established before the review commenced to enhance transparency and methodological rigor. Formal prospective registries such as PROSPERO do not currently accept protocols for scoping reviews; in lieu of registration, the risk of bias was minimised through *a priori* definition of PCC-based eligibility criteria, use of a piloted standardised data-charting form, and independent dual screening with a fourth-reviewer arbitration process for unresolved disagreements.

### Research questions

This review was guided by the following research questions.

1. How has the evidence base regarding enamel and dentin defects in preterm-born individuals evolved over the past four decades, and what temporal or methodological trends are apparent?
2. What types of enamel and dentin defects have been reported in preterm-born individuals?
3. What pathophysiological mechanisms have been proposed, and where do these mechanisms conflict or remain unvalidated across the studies?
4. What clinical consequences are associated with enamel and dentin defects in this population?
5. What preventive or management strategies have been described?
6. What methodological inconsistencies (diagnostic criteria, gestational age cut-offs, dentition assessed) limit comparability across studies, and how should future research standardise these?

### Search strategy

A comprehensive literature search was conducted in PubMed, Scopus, and Web of Science to identify relevant studies published between January 1985 and July 2026. The search period commenced in 1985 because this corresponds to the earliest published evidence examining developmental defects of enamel and dentin in individuals born preterm, thereby enabling the evolution of evidence across changing neonatal care practices to be mapped comprehensively. Following peer review, an updated literature search was performed through July 2026 using the same search strategy and eligibility criteria to ensure the inclusion of the most recent evidence prior to manuscript revision. The search strategy was developed using three core concepts: preterm birth, developmental defects in dental hard tissues, and oral health outcomes. PubMed, Scopus, and Web of Science were selected because they provide the broadest multidisciplinary coverage of dental, paediatric, and general biomedical literature relevant to this topic. Embase, the Cochrane Library, CINAHL, and Dentistry & Oral Sciences Source were not searched because of resource and access constraints, which is acknowledged as a limitation below.

Search terms related to preterm birth included “preterm birth,” “prematurity,” “preterm infant,” “very low birth weight,” and “extremely low birth weight.” These terms were combined with keywords related to enamel and dentin defects, including “enamel hypoplasia,” “enamel hypomineralization,” “developmental defects of enamel,” “dentin defects,” “amelogenesis,” and “dentinogenesis,” as well as oral health outcome terms such as “dental caries,” “tooth development,” “primary dentition,” and “permanent dentition.” Boolean operators, truncations, and database-specific controlled vocabulary terms (including MeSH terms, where applicable) were used to optimise retrieval. The complete search strategy for each database is presented in Table 1.

**Table 1:** Complete search strategy for pubMed, scopus, and web of science.

Database	Complete search strategy	Search limits
PubMed (MEDLINE)	((“Preterm Birth”[Mesh] OR “Premature Birth” OR prematurity OR “preterm birth” OR “preterm infant” OR “preterm infants” OR “premature infant” OR “Low Birth Weight”[Mesh] OR “low birth weight” OR LBW OR “Very Low Birth Weight” OR VLBW OR “Extremely Low Birth Weight” OR ELBW) AND (“Dental Enamel Hypoplasia”[Mesh] OR “enamel hypoplasia” OR “enamel hypomineralization” OR “developmental defects of enamel” OR “developmental enamel defects” OR “developmental dental defects” OR “dental hard tissue defects” OR “dentin defects” OR “developmental defects of dentin” OR dentinogenesis OR amelogenesis OR “molar incisor hypomineralization” OR MIH OR DDE) AND (“Dental Caries”[Mesh] OR “dental caries” OR “tooth development” OR odontogenesis OR “primary dentition” OR “permanent dentition” OR “tooth mineralization” OR “dental hypersensitivity”))*	Humans; English language; Publication date: January 1985–July 2026
Scopus	TITLE-ABS-KEY (“preterm birth” OR prematurity OR “premature birth” OR “preterm infant” OR “premature infant” OR “low birth weight” OR LBW OR “very low birth weight” OR VLBW OR “extremely low birth weight” OR ELBW) AND TITLE-ABS-KEY (“enamel hypoplasia” OR “enamel hypomineralization” OR “developmental defects of enamel” OR “developmental enamel defects” OR “developmental dental defects” OR “developmental defects of dentin” OR “dental hard tissue defects” OR “dentin defects” OR dentinogenesis OR amelogenesis OR “molar incisor hypomineralization” OR MIH OR DDE) AND TITLE-ABS-KEY (“dental caries” OR “tooth development” OR odontogenesis OR “primary dentition” OR “permanent dentition” OR “tooth mineralization” OR hypersensitivity)*	English language; Articles; Publication years: January 1985–July 2026
Web of Science Core Collection	*TS* *=* ((“preterm birth” OR prematurity OR “premature birth” OR “preterm infant” OR “premature infant” OR “low birth weight” OR LBW OR “very low birth weight” OR VLBW OR “extremely low birth weight” OR ELBW) AND (“enamel hypoplasia” OR “enamel hypomineralization” OR “developmental defects of enamel” OR “developmental enamel defects” OR “developmental dental defects” OR “developmental defects of dentin” OR “dental hard tissue defects” OR “dentin defects” OR dentinogenesis OR amelogenesis OR “molar incisor hypomineralization” OR MIH OR DDE) AND (“dental caries” OR “tooth development” OR odontogenesis OR “primary dentition” OR “permanent dentition” OR “tooth mineralization” OR hypersensitivity))*	English language; Articles and Review Articles; Publication years: January 1985–July 2026

**Additional Search Strategy:** Following the primary electronic database search, the reference lists of all included studies and relevant review articles were manually screened to identify additional eligible publications. After peer review, the electronic search was updated using the same search strategy through July 2026, and any newly identified studies meeting the predefined Population–Concept–Context (PCC) eligibility criteria were incorporated into the final review. Grey literature, including conference proceedings, dissertations, theses, and trial registries, was not systematically searched because of resource and access constraints. Consequently, unpublished null or negative findings may not have been captured, potentially introducing publication bias and overestimating the apparent association between prematurity and enamel or dentin defects.

* Search terms within each concept were combined using the Boolean operator OR, and the three concepts were combined using the Boolean operator AND. Search syntax was adapted as required for each database.

The reference lists of all included studies and relevant review articles were manually screened to identify additional eligible publications. Grey literature was not systematically searched, which is acknowledged as a limitation of this review. Sources such as theses, conference proceedings, and trial or study registries were not captured, which may have disproportionately excluded unpublished null or negative findings, potentially inflating the apparent strength of the association between prematurity and enamel/dentin defects reported here. The updated search identified additional eligible studies published between 2022 and 2025, which were incorporated into the final synthesis to ensure that the review reflects the most current evidence available until July 2026.

### Eligibility criteria

The eligibility criteria were developed using the Population–Concept–Context (PCC) framework recommended by the Joanna Briggs Institute (13).

### Population

Individuals born preterm (<37 completed weeks of gestation), including very low birth weight (VLBW), and extremely low birth weight (ELBW) subgroups.

### Concept

Developmental defects affecting the enamel and/or dentin, including enamel hypoplasia, enamel hypomineralisation, molar-incisor hypomineralisation, dentin mineralisation abnormalities, structural dentin defects, and associated clinical outcomes such as dental caries, hypersensitivity, and restorative challenges.

### Context

Clinical, community-based, epidemiological, and laboratory settings involving primary or permanent dentition.

Studies were included if they met the following criteria:

- Evaluated enamel and/or dentin outcomes in individuals born preterm;
- Reported original primary research findings;
- Were published in English between 1985 and July 2026; and
- Provided sufficient methodological and outcome data for extraction.

Studies were excluded if they met the following criteria:

- Did not investigate dental hard tissue outcomes in the preterm population
- Focused primarily on syndromic, genetic, or developmental disorders unrelated to prematurity.
- Were conference abstracts, editorials, commentaries, or opinion papers lacking primary data; or
- Insufficient methodological information was provided.

### Study selection

**Figure 2:**
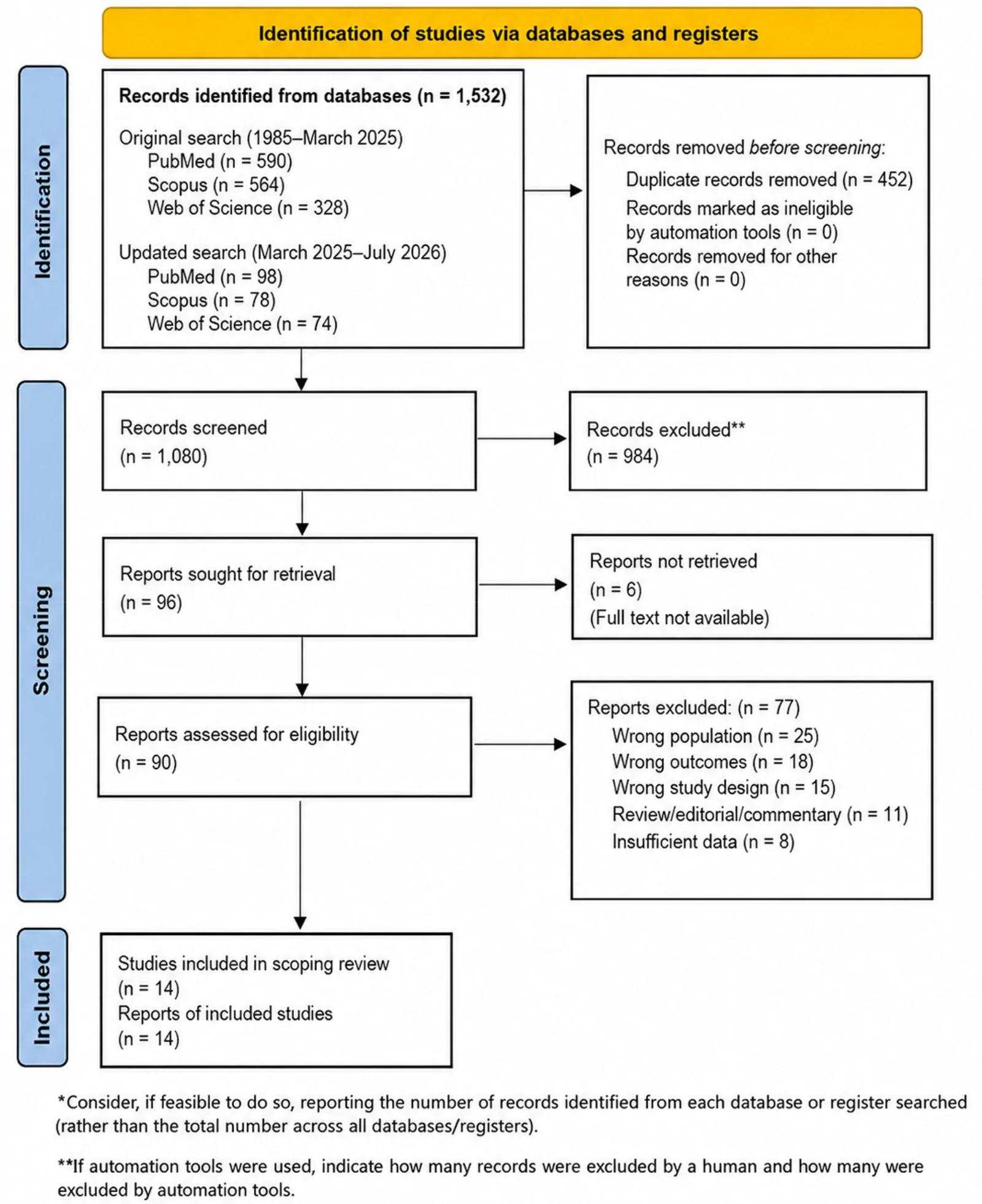
PRISMA-ScR flow diagram showing study selection process.

All identified records were imported into reference management software, and duplicate records were removed before screening. Study selection was performed independently by three reviewers (S.B., S.S., and S.U.) in two stages:

Initially, the titles and abstracts were screened against the predefined eligibility criteria. Studies considered potentially relevant were subsequently assessed through full-text reviews. The reasons for exclusion during the full-text screening stage were documented and are presented in the PRISMA-ScR flow diagram (Figure 2).

Disagreements between the reviewers were resolved through discussion and consensus. In cases where consensus could not be achieved, a fourth reviewer (M.R.) was consulted.

### Data charting and synthesis

A standardised data-charting form was developed *a priori* and piloted on a subset of studies to ensure consistency and completeness. Data extracted from each study included the author, year of publication, country, study design, study setting, sample characteristics, gestational age, birth weight, age at assessment, dentition evaluated, comparator groups, diagnostic criteria, dental outcomes assessed, principal findings, and reported risk or modifying factors (Table 2).

**Table 2:** Characteristics and principal findings of the studies included in the scoping review.

Author, Year, Country	Study design	Sample size	Study group	Comparison group	Dentition assessed	Outcomes assessed	Key findings
Seow et al., (4), Australia	Cross-sectional	129 (90 preterm/LBW, 39 term)	Preterm children (≤32 weeks GA)	Full-term children	Primary	Enamel hypoplasia	Significantly higher prevalence and severity of enamel hypoplasia in preterm children: a dose-response relationship with decreasing gestational age and birth weight.
Fearne et al., (5), UK	Observational cohort	203 (110 LBW, 93 controls)	LBW children (<2,000 g)	Normal birth weight children	Primary	Enamel defects	Increased enamel defects in incisors and molars are associated with LBW and the severity of neonatal illness.
Lai et al., (6), Australia	Case-control, longitudinal	102 (51 VLBW, 51 controls)	VLBW children	Full-term controls	Primary	Enamel hypoplasia, dental caries	Higher frequency of enamel defects and increased caries prevalence in preterm children.
Jälevik & Norén, (10), Sweden	Morphological/histological	216 teeth	Permanent molars with hypomineralisation	Sound permanent molars	Permanent	Dentin porosity	Increased dentin porosity beneath hypomineralised enamel in affected molars.
Aine et al., (11), Finland	Longitudinal	147 (74 preterm, 73 term)	Preterm children	Full-term peers	Primary and permanent	Enamel defects	Enamel defects originating in primary teeth persist in permanent dentition.
Cruvinel et al., (12), Brazil	Cross-sectional	190 (95 preterm, 95 term)	Preterm children	Full-term children	Primary	Dentin sensitivity, enamel defects	Greater dentin sensitivity and structural fragility in preterm children.
Nelson et al., (7), USA	Cross-sectional	157 (89 VLBW, 68 controls)	Adolescents with VLBW history	Full-term adolescents	Permanent	Enamel defects, dental caries	Enamel defects remain prevalent during adolescence, with increased caries susceptibility.
Sabel et al., (13), Sweden	Histological analysis	Not applicable (histological thesis/monograph)	Preterm children	Full-term children	Primary	Dentin mineralisation, tubule morphology	Reduced dentin mineralisation and altered dentinal tubule morphology in preterm teeth.
Rythén et al., (14), Sweden	Clinical and histological	157 (89 extremely preterm, 68 controls)	Extremely preterm children	Full-term controls	Primary and permanent	Dentin hardness, enamel defects	Reduced dentin hardness and increased prevalence of enamel defects are associated with prematurity.
Paulsson et al., (15), Sweden	Cross-sectional	115 (36 EPT, 37 VPT, 42 term)	Extremely and very preterm children	Term-born peers	Permanent	Developmental enamel defects	Prematurity is independently associated with developmental enamel defects in the permanent dentition.
Halperson et al., (31), Israel	Retrospective cohort	107 (52 preterm, 55 term)	Children born <32 weeks GA (1–4 years)	Full-term children	Primary	Developmental defects of enamel (DDE)	DDE occurred in **44%** of preterm children vs. **11%** of term children (OR 6.47). Respiratory distress syndrome, surfactant therapy, and prolonged mechanical ventilation were independently associated with DDE, supporting both systemic and local (intubation-related) mechanisms.
Lunardelli et al., 2023, Brazil*	Cross-sectional	655	Six-year-old schoolchildren; prematurity analysed as an exposure variable	Term-born peers within cohort	Primary	Enamel defects (opacities, hypoplasia) and prenatal/neonatal/postnatal risk factors	The overall DDE prevalence was **44.0%**. Prematurity remained associated with enamel defects after adjusting for prenatal, neonatal, and postnatal factors.
Park (Kang) et al., (33), Republic of Korea	Prospective cohort	118 preterm infants	Extremely, very and moderate-to-late preterm infants	None	Primary	Enamel defects (PDDE severity index)	Gestational age <28 weeks, birth weight <1,000 g, and low Apgar scores independently predicted DDE. Bronchopulmonary dysplasia, intraventricular haemorrhage and necrotising enterocolitis were associated with greater defect severity.
Toscan et al., (34), Brazil†	Cross-sectional	93	Very low birth weight infants	None	Primary	Developmental enamel defects (Modified DDE Index)	The prevalence of DDE was **67%** (95% CI 57%–76%), with enamel hypoplasia predominantly affecting the bilateral maxillary incisors. The bilateral distribution suggests a predominantly systemic, rather than a local, intubation-related mechanism.

GA, gestational age; LBW, low birth weight; VLBW, very low birth weight; NICU, neonatal intensive care unit; NR, not reported.

Narrative reviews and other secondary evidence sources were used to provide contextual information but were not considered primary evidence during the data synthesis.

Bold values indicate sample sizes of the included studies.

Data extraction was performed by one reviewer (S.B.) and independently verified by two other reviewers (S.S. and S.U.). Any discrepancies were resolved through discussion and consensus.

The extracted data were descriptively synthesised using narrative and thematic approaches. The findings were organised into the following domains: (1) enamel defects associated with prematurity, (2) dentin alterations associated with prematurity, (3) proposed biological and clinical mechanisms, (4) clinical implications, and (5) evidence gaps and future research priorities. Quantitative synthesis was not performed because of substantial heterogeneity in the study design, outcome definitions, and assessment methods.

### Critical appraisal of evidence

Consistent with established scoping review methodology, a formal risk-of-bias assessment intended to determine the certainty of evidence was not undertaken, as the primary objective of this review was to map and characterise the available literature rather than evaluate intervention effectiveness or generate pooled effect estimates (9, 10). Nevertheless, in response to reviewer feedback, a methodological quality appraisal of the included studies was conducted using the design-specific Joanna Briggs Institute (JBI) critical appraisal checklists, applying the checklist most appropriate for each study design (cross-sectional, cohort, case-control, and an adapted checklist for histological/morphological studies). The results of this appraisal are presented in Table 3. Overall, the included studies demonstrated moderate to high methodological quality; however, common limitations included predominantly observational study designs, relatively small sample sizes, heterogeneity in diagnostic criteria for developmental defects of enamel and dentin, infrequent reporting of examiner calibration or blinding, and limited adjustment for important neonatal confounders, such as respiratory distress syndrome, bronchopulmonary dysplasia, prolonged mechanical ventilation, nutritional deficiencies, and medication exposure. These methodological limitations should be considered when interpreting the findings of this review.

**Table 3:** Joanna Briggs Institute (JBI) Critical Appraisal of the Included Studies.

Study	Study design	JBI checklist used	Overall methodological quality	Main methodological limitations
Seow et al., (4)	Cross-sectional	JBI Critical Appraisal Checklist for Analytical Cross-Sectional Studies	Moderate	Limited adjustment for confounders; examiner blinding not reported; single-centre study.
Fearne et al., (5)	Cohort	JBI Critical Appraisal Checklist for Cohort Studies	Moderate	Limited control for neonatal confounders; follow-up procedures insufficiently described.
Lai et al., (6)	Case-control (longitudinal)	JBI Critical Appraisal Checklist for Case-Control Studies	Moderate	Matching described, but residual confounding and examiner calibration not reported.
Jälevik & Norén, (10)	Histological/Morphological	Adapted JBI Checklist for Analytical Observational Studies*	Moderate	Small sample; laboratory-based findings with limited clinical generalisability.
Aine et al., (11)	Longitudinal cohort	JBI Critical Appraisal Checklist for Cohort Studies	High	Appropriate comparison group; limited adjustment for neonatal interventions.
Cruvinel et al., (12)	Cross-sectional	JBI Cross-Sectional Checklist	Moderate	Cross-sectional design limits causal inference; examiner calibration incompletely described.
Nelson et al., (7)	Cross-sectional	JBI Cross-Sectional Checklist	Moderate	Potential selection bias; residual confounding.
Sabel et al., (13)	Histological analysis	Adapted JBI Checklist for Analytical Observational Studies*	Moderate	Experimental histological design; limited clinical applicability; no patient-level adjustment.
Rythén et al., (14)	Clinical and histological	Adapted JBI Checklist	Moderate	Small sample size; examiner blinding not clearly reported.
Paulsson et al., (15)	Cross-sectional	JBI Cross-Sectional Checklist	High	Appropriate comparison group; limited adjustment for neonatal comorbidities.
Halperson et al., (31)	Retrospective cohort	JBI Cohort Checklist	High	Good comparator group and multivariable analysis; retrospective design.
Lunardelli et al., (32)^†^	Cross-sectional	JBI Cross-Sectional Checklist	Moderate	Prematurity analysed as an exposure rather than the primary study population; cross-sectional design.
Park et al., (33)	Prospective cohort	JBI Cohort Checklist	High	No full-term comparison group; otherwise well-designed prospective cohort with multivariable analyses.
Toscan et al., (34)^‡^	Cross-sectional	JBI Cross-Sectional Checklist	Moderate	Single-group prevalence study without comparator; limited external validity.

Histological and morphological studies were appraised using the most appropriate adapted JBI analytical observational criteria because no dedicated JBI checklist exists for these study designs.

† Lunardelli et al. 2023 and ^‡^Toscan et al. 2025 are newly added references (31–34); see updated reference list.

### Ethical considerations

As this review involved the analysis of previously published literature and did not include human participants, animals, or identifiable personal data, ethical approval and informed consent were not required for this study.

## Discussion

This scoping review mapped the available evidence regarding developmental defects of the enamel and dentin in individuals born preterm and identified key knowledge gaps within this field. Overall, the findings indicate a consistent association between prematurity and developmental defects affecting dental hard tissues, particularly enamel hypoplasia and hypomineralisation. Although the included studies varied considerably in terms of design, diagnostic criteria, and outcome assessment, the direction of the evidence was largely consistent across different populations and settings. Enamel defects were the most frequently investigated outcomes, whereas dentin-related alterations were reported less frequently and remain comparatively underexplored (16–19, 24–26).

The predominance of enamel defects among preterm-born individuals is biologically plausible, given the sensitivity of amelogenesis to systemic disturbances during critical periods of tooth development. Ameloblasts are highly specialised cells that lack the capacity for regeneration following injury; therefore, disruptions during the secretory or maturation stages of enamel formation may result in permanent structural defects (16, 17, 22). Several studies included in this review reported a higher prevalence and severity of enamel hypoplasia and hypomineralisation in children born preterm, particularly those with a lower gestational age and birth weight (17, 19, 24–26).

An important observation emerging from this review is that developmental disturbances associated with prematurity may extend beyond primary dentition. Longitudinal studies have reported the persistence of enamel defects in the permanent dentition, suggesting that the effects of early life systemic insults may have long-term consequences for oral health (17, 25, 26). These defects are clinically significant because compromised enamel structures may facilitate plaque retention, bacterial colonisation, and acid penetration, thereby increasing susceptibility to dental caries and restorative complications (20, 21, 23, 29). Consequently, the oral health burden associated with prematurity may persist throughout childhood and adolescence, emphasising the importance of long-term dental surveillance in this population group (20–22).

Compared with enamel defects, there is considerably less evidence regarding dentin abnormalities in preterm-born individuals. The limited evidence suggests that prematurity may influence dentin development through alterations in mineralisation and microstructural organisation. Histological and clinical investigations have reported changes in dentin hardness, mineralisation, and structural characteristics, indicating that the effects of prematurity on odontogenesis may not be confined solely to enamel formation. However, the small number of studies and variability in assessment methods preclude definitive conclusions regarding the nature and extent of dentin involvement in these cases (16, 17, 22). Nevertheless, dentin abnormalities may contribute to hypersensitivity, structural fragility, accelerated wear, and restorative challenges, highlighting the need for further investigation.

The evidence synthesised in this review suggests that the relationship between prematurity and developmental dental defects is multifactorial. Proposed mechanisms include perinatal hypoxia, nutritional deficiencies, systemic inflammation, metabolic disturbances, and neonatal intensive care–related exposure. These factors frequently coexist during the neonatal period and may collectively interfere with normal amelogenesis and dentinogenesis (22, 27, 28). Consequently, gestational age and birth weight alone may not fully explain the observed variations in dental outcomes (24, 25).

Importantly, neonatal intensive care–related factors may independently affect dental hard tissue development. Prolonged endotracheal intubation other respiratory interventions have been implicated in developmental disturbances affecting the dentition of preterm infants (27). The absence of adequate adjustment for neonatal factors in many observational studies limits the extent to which the observed associations can be attributed to prematurity *per se*, rather than to co-occurring neonatal interventions and morbidities.

The potential contribution of neonatal illness and intensive care–related exposure therefore warrants particular attention. Several studies have suggested that the severity of neonatal complications and their management may influence the occurrence of developmental dental defects (17, 19). As advances in neonatal care continue to improve survival among extremely preterm infants, understanding the long-term oral consequences of prematurity and associated medical management is becoming increasingly important.

From a clinical perspective, the findings of this review support the incorporation of gestational history into routine dental assessments. Early identification of preterm children may facilitate targeted preventive strategies, individualised recall schedules, and timely management of emerging defects (28). Greater collaboration between neonatologists, paediatricians, and paediatric dentists may improve the continuity of care and contribute to improved long-term oral health outcomes.

The clinical management of developmental enamel defects should consider the type, severity, and functional consequences of the defect. Children with hypoplastic or hypomineralised enamel may require enhanced preventive measures and appropriate restorative interventions to minimise the risk of caries, sensitivity, and structural breakdown (20, 30).

Several evidence gaps have been identified. First, substantial heterogeneity exists in the diagnostic criteria used to assess developmental defects, limiting comparability across studies. The clinical diagnosis of enamel defects itself may be challenging because of variations in terminology and diagnostic approaches (16, 22). Second, most available evidence is derived from cross-sectional investigations, which restricts our understanding of the natural history and long-term progression of these defects. Third, dentin-specific outcomes remain inadequately investigated compared with those of enamel defects.

Overall, this review highlights a consistent but methodologically diverse body of evidence suggesting an association between preterm birth and developmental defects in enamel and dentin (18). While enamel defects have been relatively well documented, important uncertainties remain regarding dentin pathology, underlying biological mechanisms, and long-term clinical outcomes. Future longitudinal and multicentre studies employing standardised diagnostic frameworks are needed to strengthen the evidence base and support the development of evidence-informed preventive and therapeutic strategies for this vulnerable population group.

## Conclusion

This scoping review identified a consistent body of evidence suggesting an association between preterm birth and developmental defects of the enamel and dentin, particularly enamel hypoplasia and hypomineralisation. Lower gestational age and very low birth weight appear to be important factors associated with the increased prevalence and severity of these defects. The available literature suggests that multiple biological and clinical factors, including perinatal hypoxia, nutritional deficiencies, systemic inflammation, and neonatal intensive care–related exposures, may contribute to the disruption of normal odontogenesis.

Despite the increasing recognition of the oral health consequences of prematurity, important knowledge gaps remain. Evidence regarding dentin-specific alterations is limited, long-term longitudinal data are scarce, and preventive or therapeutic interventions tailored to preterm individuals have not been adequately investigated. Future research should prioritise standardised diagnostic criteria, prospective longitudinal study designs, and mechanistic investigations to strengthen the understanding of developmental dental defects associated with prematurity. Clinically, incorporating gestational history into routine dental assessments and implementing early preventive surveillance strategies may facilitate the timely identification and management of at-risk children and contribute to improved long-term oral health outcomes.
